# Effect of Some Substituents Increasing the Solubility of Zn(II) and Al(III) Phthalocyanines on Their Photophysical Properties

**DOI:** 10.1155/2014/952632

**Published:** 2014-09-11

**Authors:** A. A. Chernonosov, E. A. Ermilov, B. Röder, L. I. Solovyova, O. S. Fedorova

**Affiliations:** ^1^Institute of Chemical Biology and Fundamental Medicine, Siberian Branch of the Russian Academy of Sciences, Lavrentiev Avenue 8, Novosibirsk 630090, Russia; ^2^Institut für Physik, Photobiophysik, Humboldt-Universität zu Berlin, Newtonstraße 15, 12489 Berlin, Germany; ^3^Institute of Organic Intermediates and Dyes, B. Sadovaya 1/4, Moscow 123995, Russia

## Abstract

Water solubility of phthalocyanines (Pcs) usually increases by the introduction of charged or carboxy substituents in the peripheral positions of the macrocycle. As a result, such structural changes influence their photophysical and photochemical properties as photosensitizers. Phthalocyanines substituted with four or eight terminal carboxyl groups and having in some cases additional eight positive charges (water soluble phthalocyanines) were studied in order to evaluate the spectroscopic and photophysical effects of these side residues on the chromophore properties. The quantum yield of singlet oxygen (^1^O_2_) generation, the triplet-triplet absorption, and the transient absorption spectra were measured and linked to the structure of the substituents. It was shown that charged substituents did not change the quantum yields of ^1^O_2_ generation but decrease its lifetimes. The introduction of the charged substituents not only increases the water solubility but also significantly changes absorption, fluorescence, and transient absorption spectra of water soluble Pcs.

## 1. Introduction

Phthalocyanines are purely synthetic analogues of porphyrins. They have a great potential as photosensitizers in photodynamic therapy (PDT) for the treatment of malignancies and other diseases [1–4]. As photosensitizers, Pcs have the advantage of high molar absorption [5, 6], resistance to chemical and photochemical degradation, and long lifetimes of ^1^O_2_ with high quantum yields [7]. Moreover, Pcs have absorption and emission in the range of 660–750 nm, making them sensitive to the light, which can penetrate deeply into living tissues [8]. Propensity to aggregation due to molecular stacking resulting in low quantum yields of ^1^O_2_ [9, 10] and limiting solubility in aqueous media is the disadvantage of these dyes.

**Figure 1:**
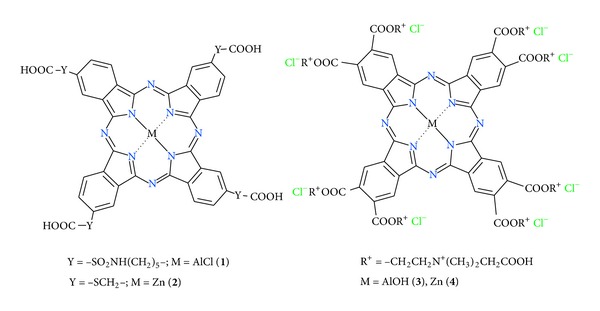
Structures of the Pc complexes** 1**–**4**.

The chemical structure of Pcs can be modified by the introduction of substituents in the peripheral positions of the tetraaza isoindole macrocycle [11], as well as by the coordination of metal ions with the central nitrogen atoms and the addition of axial ligands in the fifth and sixth coordinative positions of such molecules [12, 13]. Primary goal of such modifications of Pcs is to tune the water solubility and aggregation [14, 15] without significant changes in the photophysical properties. Introduction of several identical groups, for example, carboxylic groups, does not break significantly the symmetry of the Pc molecules but usually reduces their aggregation. In this work, we report the spectroscopic and photophysical characteristics of several new Pcs derivatives that can potentially serve as photosensitizers for PDT with favorable water solubility. We examined Al(III) tetra-4-carboxymethyl sulfanyl (**1**), Zn(II) tetra-4-carboxyhexylsulfamidophthalocyanine (**2**) having terminal carboxyl groups as well as Al(III) and Zn(II) octakis-4,5-[2-(N-carboxymethyl-N,N-dimethylammonium)ethoxycarbonyl] phthalocyanine octachlorides (**3** and** 4**) having eight charged substituents (Figure 1) in order to evaluate the spectroscopic and photophysical effects of these side residues on the chromophore properties.

The quantum yield of ^1^O_2_ generation, the measurements of the triplet-triplet absorption, and the picosecond transient absorption spectra (ps-TAS), which are crucial in determining the feasibility of using these dyes for PDT, are reported and linked to the structure of the substituents.

## 2. Materials and Methods

### 2.1. Chemicals and Reagents

Dimethylformamide (DMF; Sigma, Germany), D_2_O, and double-distilled water were used as solvents.

### 2.2. Al(III) Tetra-4-carboxymethylsulfanylphthalocyanine (**1**)

This compound has been prepared by analogy with [16]. The yield of compound** 1 **was about 30%. UV-vis, *λ*_max⁡_, nm (DMF): 680, 613, 353 (relative intensity 1 : 0.2 : 0.35); (water : ethanol, 1 : 1): 693, 652 sh, 618 sh, 349 (relative intensity 0.85 : 0.65 : 0.5 : 1).

### 2.3. Zn(II) Tetra-4-carboxyhexylsulphamidophthalocyanine (**2**)

This compound has been prepared by analogy with [16]. The yield of compound** 1** was about 30%. UV-vis, *λ*_max⁡_, nm (DMF): 690, 622, 363 (relative intensity 1 : 0.22 : 0.44); (water : ethanol, 1 : 1): 682 sh, 637, 344 (relative intensity 0.59 : 0.78 : 0.85).

### 2.4. Hydroxyaluminum Octakis-4,5-[2-(N-carboxymethyl-N,N-dimethylammonium)ethoxycarbonyl] Phthalocyanine Octachloride (**3**)

To a solution of 0.1 mmol hydroxyaluminum octakis-4, 5-(2-chloroethoxycarbonyl) phthalocyanine [17] in 1.5 mL anhydrous N-methylpyrrolidone 1.8 mmol N,N-dimethylglycine and catalytic amount of sodium iodide were added, and reaction mixture was stirred 6 h at 105–110°C, cooled, and poured in the mixture of petroleum ether-benzene (3 : 1); solvent was decanted; the precipitate was washed with benzene and ether and then dried in a vacuum desiccator over phosphorus pentoxide. The yield of compound** 3** was about 90%. UV-vis, *λ*_max⁡_, nm (water): 690, 652, 350 (relative intensity 0.95 : 0.92 : 1); (ethanol): 691, 682, 617, 352 (relative intensity 1.97 : 1.95 : 0.72 : 1).

### 2.5. Zinc Octakis-4,5-[2-(N-carboxymethyl-N,N-dimethylammonium)ethoxycarbonyl] Phthalocyanine Octachloride (**4**)

This was prepared analogously from zinc octakis-4,5-(2-chloroethoxycarbonyl) phthalocyanine [17] with yield of 92%. UV-vis, *λ*_max⁡_, nm (water): 685 sh, 641, 345 (relative intensity 0.74 : 1.07 : 1); (ethanol): 676, 643, 344 (relative intensity 1 : 1.08 : 10).

Compounds** 1** and** 2** are soluble in polar solvents especially DMSO and DMF as well as aqueous acetonitrile and alcohols. Compounds** 3** and** 4** are soluble in water, concentrated mineral acids, and their aqueous solutions, alcohols, and polar aprotic solvents. UV-vis absorption spectra of their aqueous solutions show an aggregation, to a lesser extent in the case of aluminum complex** 3**.

### 2.6. Absorption and Steady-State Fluorescence

The ground state absorption spectra were recorded using a spectrophotometer Shimadzu UV-2501PC at room temperature. The steady-state fluorescence spectra were measured using 1 cm × 1 cm quartz cells with a combination of a cw-Xenon lamp (XBO 150) and a monochromator (Lot-Oriel, bandwidth 10 nm) for excitation and a polychromator equipped with a cooled CCD matrix as a detector system (Lot-Oriel, Instaspec IV) [18].

### 2.7. Time-Resolved Singlet Oxygen Luminescence Measurement

These measurements were carried out using a nanosecond Nd, YAG laser (BM Industries, Evry Cedex, France) coupled with an optical parametric oscillator (BMI). The ^1^O_2_ emission was detected using a liquid nitrogen cooled Ge-diode (North Coast, Inc., Santa Rosa, CA). For wavelength selection, a combination of a silicon filter and an interference filter with the center wavelength of 1270 nm was placed in front of the diode [19]. The optical density of the sample was adjusted to 0.2 at the excitation wavelengths in the 600–680 nm region. Tetraphenylporphyrin served as a reference standard for the determination of the singlet oxygen quantum yield (Φ_^1^O_2__) with value 0.75 [20].

### 2.8. Laser Flash Photolysis

In the triplet-triplet absorption measurements, the samples were excited using the same instrumentation as described above. A continuous wave monitoring beam was formed by light from XBO lamp that passed through a monochromator tuned to 488 nm. The beam traversed through the solutions of studied compounds perpendicular to the excitation beam. The intensity of the monitoring beam was measured using an avalanche Si-photodiode equipped with an interference filter for 488 nm. Oxygen was removed by bubbling the samples with nitrogen (purity 99,0%) for ca. 40 min at room temperature [21].

### 2.9. Picosecond Transient Absorption Spectroscopy

To measure ps-TAS, a white light continuum, generated in a cell with a D_2_O/H_2_O mixture using intense 25 ps pulses from a Nd3, YAG laser (PL 2143A, Ekspla) at 1064 nm, was used for the monitoring beam. Before passing through the sample, the monitoring beam was split to provide the reference spectrum. The transmitted as well as the reference beams were focused into two optical fibers and were recorded simultaneously at different strips on a CCD-matrix (Lot-Oriel, Instaspec IV). Tunable radiation from an OPG/OPA (Ekspla PG 401/SH, tuning range 200–2300 nm) pumped by third harmonic of the same laser was used as an excitation beam. The mechanical delay line allowed the measurement of light-induced changes of the absorption spectrum at different delays up to 15 ns after excitation. The OD of all samples was 1.0 at the maximum of the absorption band of the lowest energy. The analysis of the experimental data was performed using the compensation method [22].

## 3. Results

In the present work, the photophysical parameters of Zn(II) and Al(III) Pcs complexes** 1**–**4** containing different side residues were studied. All Pcs have terminal carboxyl groups with spacers of different lengths, and two of them (**3** and** 4**) have eight positive charges in molecule.

The chemical structures of the studied compounds are shown in Figure 1.

The tetracarboxy-phthalocyanines** 1** and** 2** are not soluble in water. However, the introduction in benzene rings of macrocycle of charged substituents leads to the increase in the solubility of the resulting compounds** 3** and** 4**. To compare photophysical properties of compounds** 1** and** 2** with water-soluble Pcs** 3** and** 4** in water-like solutions, Pcs complexes** 1** and** 2** were dissolved in 0.7% DMF in H_2_O (or in D_2_O) mixture. Such amount of DMF is appropriate to keep cells alive that is important for further testing of compounds as potential photosensitizers in PDT.

### 3.1. Absorption and Steady-State Fluorescence Spectra

**Figure 2:**
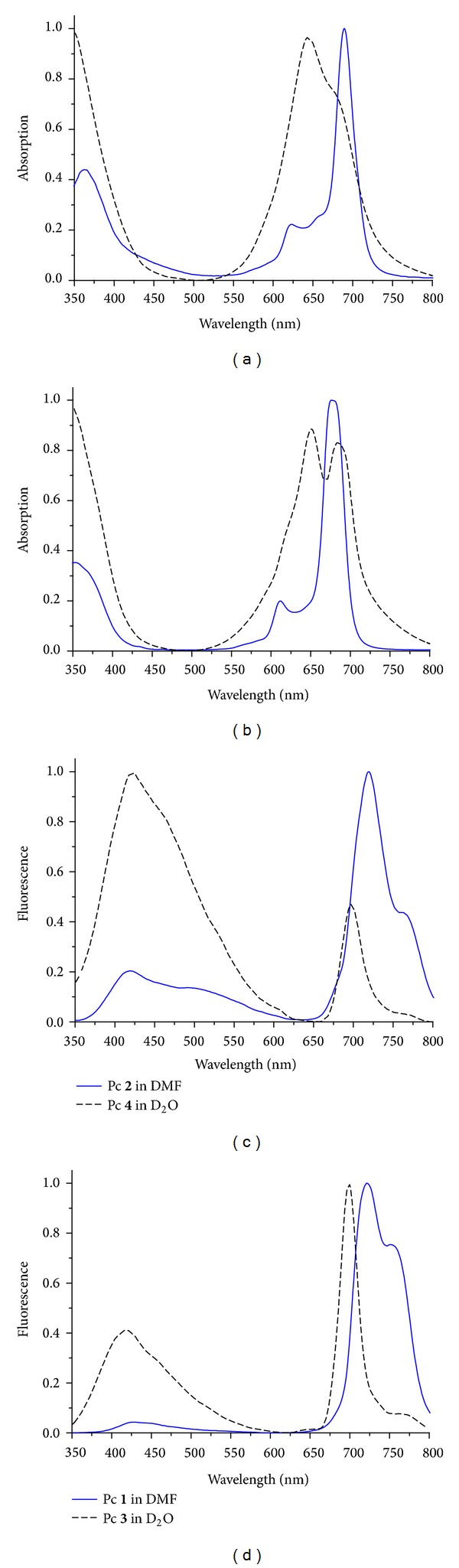
Normalized absorption spectra of the compounds containing PcZn(II)** 2**,** 4** (a) and PcAl(III)** 1**,** 3** (b). Normalized fluorescence spectra of the compounds containing PcZn(II)** 2**,** 4** (c) and PcAl(III)** 1**,** 3** (d).

The absorption spectra (see Figure 2) of the Pcs** 1** and** 2** and the containing charged residues** 3** and** 4** differ. The changes in the visible spectrum region are more significant for water-soluble Pc** 4**: the Q-band becomes broader, and its maximum shifts are from 690 nm to 650 nm. In the case of Pcs** 1** and** 3**, the Q-band at 610 nm disappears but the Q-band at 650 nm occurs. In case of Pc** 3**, the intensity of the Q-band at 690 nm decreases relatively to that at 650 nm. The Q-bands become broader for Pc** 3** as well as for Pc** 4**.

The fluorescence spectra of all compounds contain bands with a maximum in the range of 680–730 nm and a shoulder at 750–770 nm (Figure 2) as well as a maximum of different intensities in the range of 400–450 nm. Blue shift is revealed in the fluorescence spectra in the 680–730 nm region for the Pcs** 3** and** 4** in comparison to the Pcs** 1** and** 2**, respectively. Compounds** 3** and** 4** have low fluorescence intensity.

### 3.2. Singlet Oxygen Measurements

Several solvents were used in the measurement of the quantum yield of ^1^O_2_ generation due to the different solubility of the studied compounds in aqueous media. Pure DMF and 0.7% DMF in D_2_O were used for** 1** and** 2** samples; D_2_O was used for Pcs** 3** and** 4**. Tetraphenylporphyrin and tetraphenylporphyrin tetrasulfonate were used as references in organic and water solutions, respectively.

The comparison of the quantum yields of ^1^O_2_ allows finding compounds with similar behaviours (Table 1). First, the value of Φ_^1^O_2__ for compounds** 1** and** 2** in DMF and** 3** and** 4** in D_2_O was 0.22 for Pcs** 2** and** 4** containing Zn(II) and 0.11-0.12 for Pcs** 1** and** 3** with Al(III). It could mean that the increase in the solubility in water by charged side moieties does not influence significantly the Φ_^1^O_2__.

On the other hand,** 1** and** 2** are not soluble in D_2_O. The “0.7% DMF in D_2_O” solvent was used to model the behaviour of the photophysical properties of these compounds in aqueous solutions. The Φ_^1^O_2__ decreased to 0.09 for** 2** and increased up to 0.16 for** 1** compared with the results for these compounds obtained in DMF. The central metal seems to affect the solubility also. Aqueous-like solvents are more suitable for compounds** 1** and** 3** with Al(III) due to the presence of axial ligand.

The other characteristic of the photophysical properties is the lifetime of ^1^O_2_, whose well-known value in D_2_O is 60 *μ*s [23]. But for all investigated compounds, the lifetimes of ^1^O_2_ were significantly shorter: 7 *μ*s for Pcs** 3** and** 4**. The lifetime of ^1^O_2_ for Pcs** 1** and** 2** was approximately 20 *μ*s in DMF and 10 *μ*s in 0.7% DMF in D_2_O. Such difference in the lifetimes of ^1^O_2_ from the well-known values could mean that quenching of ^1^O_2_ occurs. The shortest lifetime of ^1^O_2_ was observed in the case of Pcs** 3** and** 4**, but the Φ_^1^O_2__ for these compounds was the same as that for** 1** and** 2** solved in DMF. The Pcs** 2** and** 4** were chosen for additional experiments due to their better fluorescence (compared to these with PcAl(III)) which was required in some techniques.

First, the energy transfer from the first excited triplet state *T*_1_ to molecular oxygen resulting in the generation of ^1^O_2_ was characterized. For this purpose, we used laser flash photolysis for the indirect detection of ^1^O_2_ formation via triplet lifetime measurement, which can be related to the ^1^O_2_ generation.

### 3.3. Laser Flash Photolysis

The method of laser flash photolysis was used for indirect determination of ^1^O_2_ formation via triplet lifetime measurement. This is possible because the triplet lifetimes can be related to the singlet oxygen generation. After activation of the first excited singlet state *S*_1_ via light absorption, a part of the molecules undergo transition to the first excited triplet state *T*_1_ with a quantum yield Φ_*T*_ [24]. The subsequent deactivation could proceed via phosphorescence (*k*_phos_) or nonradiative (*k*_nonrad_) transition. The fraction of triplet states deactivated via interaction with molecular oxygen is referred to as *f*_*T*_.

The triplet lifetime in the presence of oxygen *τ*_O_2__ is given by *τ*_O_2__ = (*k*_phos_+*k*_nonrad_+*k*_O_2__[O_2_])^−1^. Removal of dissolved oxygen by bubbling the solution with N_2_ leads to an increase of the triplet lifetime. In this case, the lifetime is calculated as *τ*_N_2__ = (*k*_phos_+*k*_nonrad_)^−1^.

In the presence of oxygen, the triplet lifetime of Pc** 2** in DMF was measured to be *τ*_*T*_ = 0.34 *μ*s; the lifetime of Pc** 4** in D_2_O was 6.0 *μ*s. (Table 2).

After oxygen removal, the triplet lifetimes of the compounds dissolved in the same solvents increased to *τ*_*T*_ = 52.6 *μ*s for Pc** 2** and to 8.9 *μ*s for Pc** 4** as seen in Table 2.

The value of *f*_*T*_ can be obtained finally from *τ*_O_2__ and *τ*_N_2__:

(1) fT=kO2[O2]kphos+knonrad+kO2[O2]=τN2−  τO2τN2

(see [24]). This parameter characterizes the efficiency of the energy transfer from the first excited triplet state *T*_1_ to molecular oxygen. Since in case of Pc** 2** in DMF *f*_*T*_ value is nearly unity, this means that energy transfer to the molecular oxygen is predominant and takes place almost lossless. For Pc** 4**, this value is only 0.3. However, the *f*_*T*_ value is substantially lower for Pc** 4**; the Φ_^1^O_2__ for** 2** in DMF and** 4** in D_2_O are the same. Therefore, the charged side moieties in compound** 4** have no influence on the Φ_^1^O_2__, but these moieties have influence on the photophysical properties resulting in the redistribution of the energy transfer. Also such a strong reduction of the triplet lifetime may result from the interaction between Pc core and side moieties. To determine the photophysical behavior of our compounds, more details were investigated by ps-TAS method.

### 3.4. Picosecond Transient Absorption Spectroscopy

**Figure 3:**
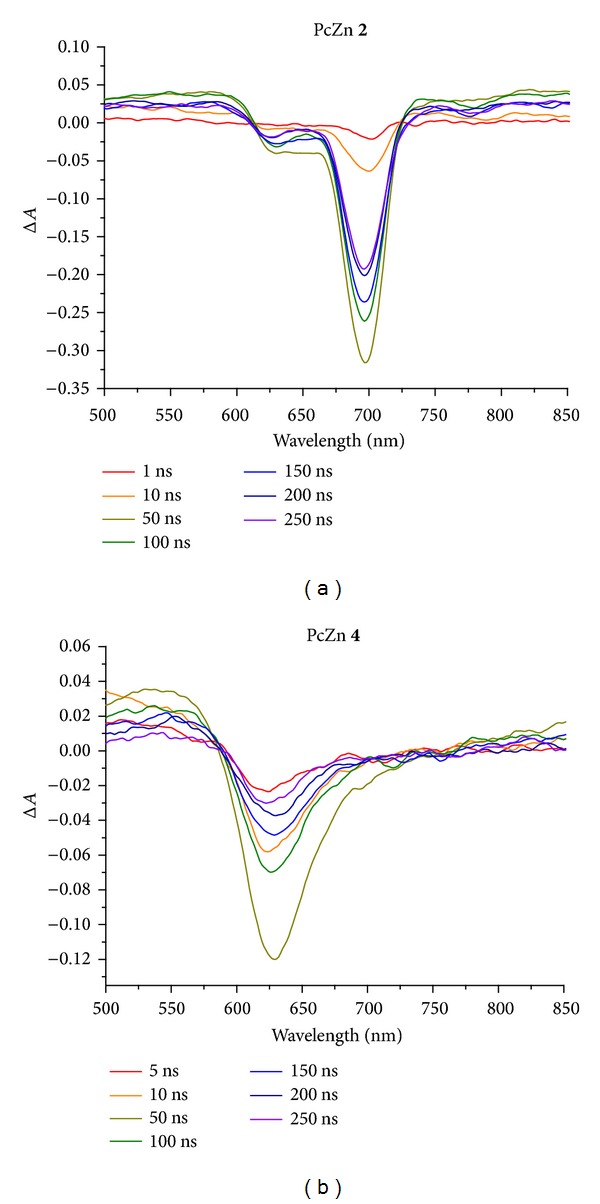
Evolution-associated difference spectra that result from a global analysis on transient absorption experiments on compounds containing** 2** and** 4**.

It was shown that the ps-TAS of Pc** 2** was different from the spectrum of Pc** 4**. Spectra of Pcs** 2** and** 4** contain only one peak at 690 and 629 nm, respectively (Figure 3). Each peak was characterized by an individual decay component.

Compound** 2** in DMF has two decay components at 690 nm with lifetimes of 177 and 2183 ps; also, Pc** 4** in D_2_O has two decay components at 629 nm with lifetimes of 57 and 286 ps (Figure 3). The amplitude ratio of the first and the second components (**A1**/**A2**) for these compounds was approximately the same: 2.58 and 2.25 for Pcs** 2** and** 4**, respectively (Table 3). It means that the shorter decay component makes primary contribution to the transient absorption spectrum. Presumably, the blue shift of the peak maximum and the significant decrease in the decay components lifetimes could be linked to the decrease of ^1^O_2_ lifetime for water-soluble Pc** 4**.

## 4. Conclusions

The introduction of the charged substituents not only increases the water solubility but also significantly changes absorption, fluorescence, and transient absorption spectra of water-soluble Pcs. It was shown that charged substituents did not change the quantum yields of ^1^O_2_ generation but they decreased its lifetimes. It could mean that the increase in the solubility in water by charged side moieties does not influence significantly the Φ_^1^O_2__, which is important for using such compound in PDT. Significant decreasing of ^1^O_2_ lifetime from the well-known value of 60 *μ*s to 7–20 *μ*s could mean that quenching of ^1^O_2_ occurs. A strong reduction of first triplet state lifetime of the Pc** 4** may result from the interaction between Pc core and side moieties. Thus, although the charged side moieties have no influence on the Φ_^1^O_2__, they influence the photophysical proprieties resulting in the redistribution of the energy transfer.

## Figures and Tables

**Table 1:** Quantum yields of ^1^O_2_ generation and lifetimes of ^1^O_2_.

Compounds	Solvent	Φ_^1^O_2__,	*T*, *μ*s
**1**	DMF	0.12	19.9 ± 0.0
**2**	DMF	0.22	22.9 ± 0.0
**1**	0.7% DMF in D_2_O	0.16	10.0 ± 0.2
**2**	0.7% DMF in D_2_O	0.09	9.3 ± 0.2
**3**	D_2_O	0.11	6.9 ± 0.5
**4**	D_2_O	0.22	6.9 ± 0.4

**Table 2:** The first triplet state lifetime of the photosensitizers obtained by laser flash photolysis in the presence and absence of the O_2_.

Compound	Solvent	*τ* with O_2_, *μ*s	*τ* without O_2_, *μ*s	Without O_2_/with O_2_	*f*_*T*_
**2**	DMF	0.34 ± 0.00	52.6 ± 0.2	152.7	0.99
**4**	D_2_O	6.0 ± 0.1	8.9 ± 0.2	1.5	0.33

**Table 3:** Decay times, amplitudes and their ratio, and the quantum yield of intersystem crossing Φ_ISC_.

Compound	Solvent	*λ*_det⁡_, nm	*τ*1, ps	**A1**, %	*τ*2, ps	**A2**, %	**A1**/**A2**	Φ_ISC_, %
**2**	DMF	690	177 ± 14	72	2183 ± 323	28	2.58	**22**
**4**	in (D_2_O)	629	57 ± 15	69	286 ± 144	31	2.25	**8**
